# A gastroenterologist’s enigma: unraveling an unusual case of melena and recurrent infective endocarditis

**DOI:** 10.1016/j.igie.2024.01.003

**Published:** 2024-01-11

**Authors:** Asaf Levartovsky, Emad Sakhnini, Marianne M. Amitai

**Affiliations:** 1 Department of Gastroenterology, Sheba Medical Center, affiliated to Tel Aviv University, Tel Aviv, Israel; 2 Department of Diagnostic Imaging, Sheba Medical Center, affiliated to Tel Aviv University, Tel Aviv, Israel

A 63-year-old man with a surgical history of a mitral valve repair and aorto-bifemoral bypass surgery was repeatedly hospitalized for the past year due to echocardiography-proven infective endocarditis. Results of his blood cultures were positive for enterobacterial bacteria (*Klebsiella pneumoniae* and *Enterococcus faecalis*). During his last hospitalization, he presented with melanotic stools and underwent upper and lower endoscopies, the results of which were unrevealing. The patient was then referred to our gastroenterology department for a small-bowel evaluation. A motorized spiral enteroscopy was performed showing a graft stent extruding from the duodenum bowel wall. Due to a high suspicion of an aortoenteric erosion or fistula, a CT angiography of the abdomen with injection of contrast material was performed; it revealed a graft stent protruding from the abdominal aorta into the duodenal bowel wall (Fig. 1). The patient eventually underwent an axillobifemoral bypass and excision of the infected graft by duodenectomy and a subsequent gastrojejunostomy. He was discharged in good condition.

**Figure 1:**
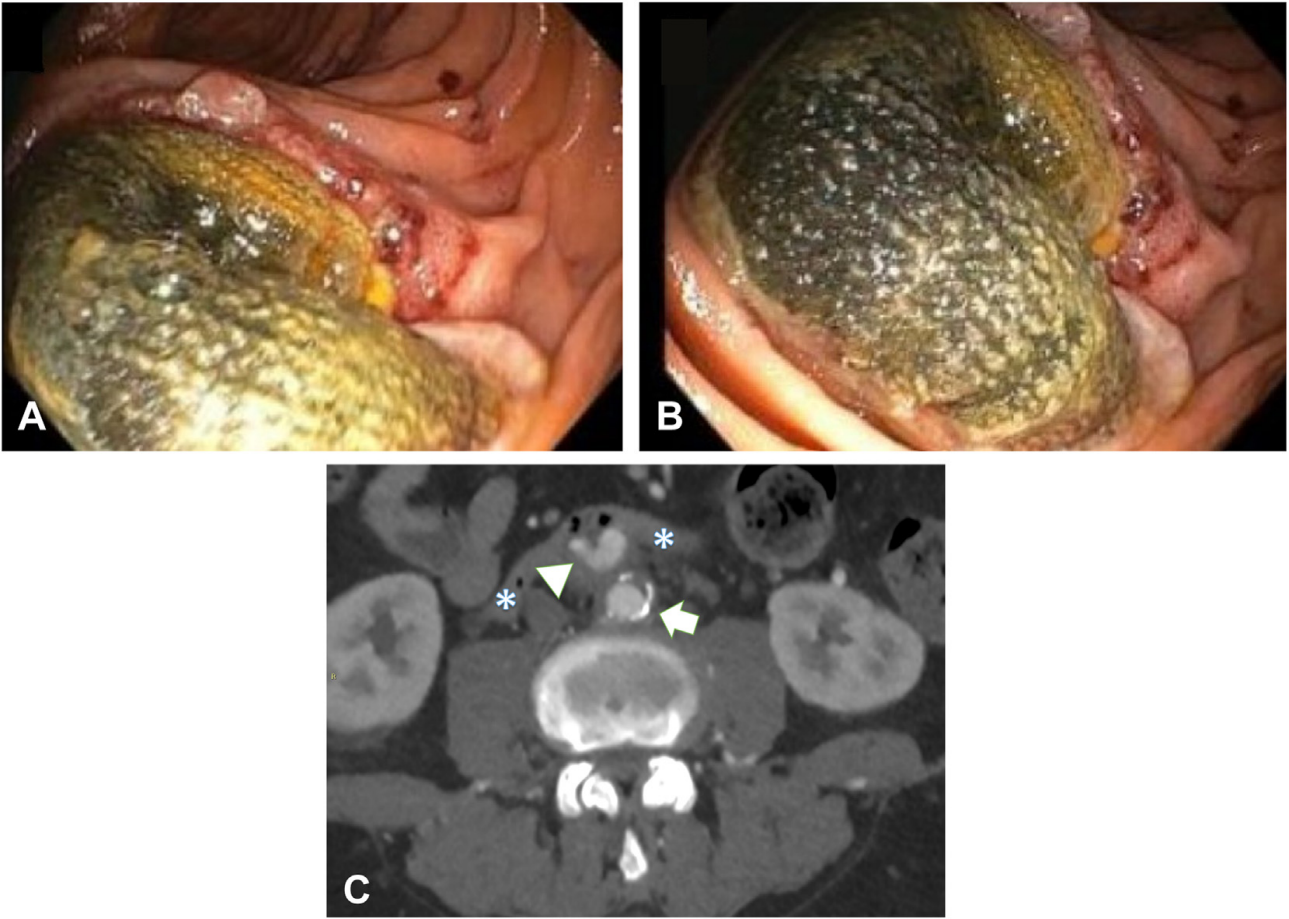
Motorized spiral Endoscopy image of the protruded stent graft. **A and B**, Motorized spiral enteroscopy showing a graft stent extruding from the duodenum bowel wall. **C**, CT angiography after injection of contrast material. The graft (*arrowhead*) is seen protruding into the duodenal bowel wall (*asterisks*). Notice the calcified aorta (*arrow*).

Secondary aortoduodenal erosion or fistula is an uncommon adverse event, occurring between the duodenum and an infected abdominal aortic surgical graft. Clinical presentation can include mild to moderate intermittent GI bleeding or even fever and recurrent bacteremia. Thus, high clinical suspicion is warranted in patients with a history of prior aortic graft placement, manifesting with recurrent bacteremia and GI bleeding.

Informed consent was obtained from the patient to publish these images.

## Disclosure

All authors disclosed no financial relationships.

